# Freeing Crop Genetics through the Open Source Seed Initiative

**DOI:** 10.1371/journal.pbio.1002441

**Published:** 2016-04-19

**Authors:** Claire H. Luby, Irwin L. Goldman

**Affiliations:** Department of Horticulture, University of Wisconsin-Madison, Madison, Wisconsin, United States of America

## Abstract

For millennia, seeds have been freely available to use for farming and plant breeding without restriction. Within the past century, however, intellectual property rights (IPRs) have threatened this tradition. In response, a movement has emerged to counter the trend toward increasing consolidation of control and ownership of plant germplasm. One effort, the Open Source Seed Initiative (OSSI, www.osseeds.org), aims to ensure access to crop genetic resources by embracing an open source mechanism that fosters exchange and innovation among farmers, plant breeders, and seed companies. Plant breeders across many sectors have taken the OSSI Pledge to create a protected commons of plant germplasm for future generations.

## Abstract 2

This Community Page article highlights the Open Source Seed Initiative, which serves as an alternative to restrictive intellectual property rights and is working to develop an open-source release mechanism for crop plant diversity, analogous to that developed for open-source software.

## Introduction

Historically, the results of scientific research have been made public to enable others to build on the results of discovery and understanding. Likewise, crop plants, long considered the common heritage of humankind, have been available for farmers and plant scientists to use in crop improvement [1]. Over the last hundred years, increasing restrictions on plant germplasm through intellectual property rights (IPR) have eroded the view of plants as a public good. In the United States, IPR restrictions may include utility patents, contracts, material transfer agreements (MTAs), trade secrets, and “bag tag” licenses (Table 1).

**Table 1:** Germplasm IPRs and their effects on plant breeding. For more detail on IPR protections (excluding Open Source), see [2].

Type of Restriction	Effect on Plant Breeding
Public Domain	Publicly accessible germplasm freely available for use. Derivatives can potentially be appropriated and protected with IPR from further use.
Open Source	A new form of publicly accessible germplasm freely available for use with the restriction that there can be no further restrictions. Derivatives are considered open source and thus similarly freely available, and all cultivars become part of an open source “protected commons.”
Plant Variety Protection Certificate (PVP)	PVP, available through the US Plant Variety Protection office since 1970, requires that cultivars be distinct, uniform, and stable. Seventeen-year certificate for new cultivars of sexually reproducing plants. Allows the plant breeder to determine who is able to sell seed and to charge royalties on use. Cultivars can be used in breeding and seed saving.
Plant Patent	Plant patents, 20-year patent available through the US Patent and Trademark Office since 1930. Covers asexually propagated crops and allows the plant breeder to charge royalties on propagation or sales of that cultivar.
Utility Patent	Utility patents, available through the US Patent and Trademark Office since the late 1980s, require that inventions be novel, non-obvious, and useful. Not historically used for plants, but many utility patents over plant cultivars and traits have been granted. Excludes others from using protected cultivars for plant breeding without permission and licensing from the holder of the patent for 20 years.
Trade Secret	Trade Secret is not a formal legal protection, but rather allows the developer to maintain control of an F1 hybrid cultivar by keeping the inbred parent lines a secret to the public. Since F1 hybrid seed will not breed true to type if saved, keeping the parent lines a secret ensures that others cannot gain access to these parent lines and cannot recreate the hybrid cultivar.
Licenses and Contracts	Outside of patent law, licenses and contracts are a form of private ordering, and restrictions are specific to the details of the agreement. These may restrict elements such as seed saving, breeding, transfer of seed to a third party, and research on the seed. They may take the form of specific agreements between parties or more generally in the form of “bag tag” licenses on seed packages.

IPRs were developed to foster innovation by protecting the rights of the inventor to claim ownership over their invention, given that the process was made public. While IPRs can be used to incentivize research and development [3], proprietary restrictions on crop plants by fewer entities consolidates the control of germplasm. This threatens the exchange of crop genetic resources necessary for innovation in plant breeding, such as developing novel cultivars to feed a growing population, perform under low-input conditions, and adapt to climate change. IPRs have also limited farmers’ and gardeners’ ability to save seeds, constrained plant breeders’ ability to use a diverse array of germplasm, and complicated the exchange of plant genetic resources around the world. At the same time, IPRs have benefited those who control crop genetic resources, which include both seed companies and universities.

**Fig 1:**
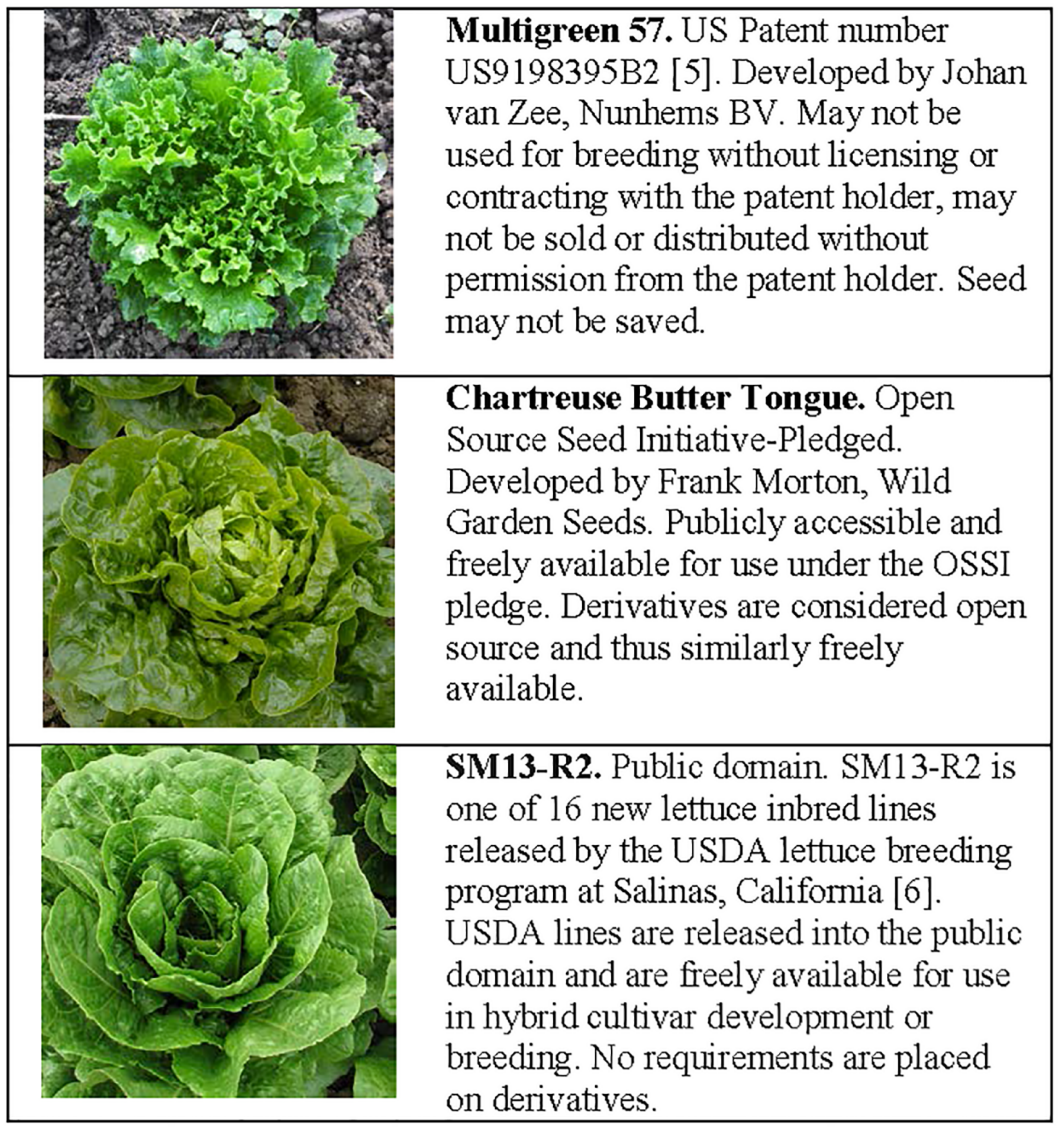
Lettuce cultivars released using three different mechanisms. Plant cultivars released into the public domain are often utilized in developing subsequent cultivars [8] that are then protected using various forms of intellectual property rights (IPR). The following lettuce cultivars are released using a variety of IPRs that allow subsequent users to utilize cultivars in future breeding in different ways. *Photo credits*: *Multigreen 57*, *Osbourne Seed Company; Chartreuse Butter Tongue*, *Karen Morton; SM13-R2*, *Jose Orozco*, *USDA/ARS*.

Besides promoting innovation, IPRs were seen as a mechanism to disseminate knowledge. However, some have questioned whether the current IPR regime encourages the exchange of information and innovation [4]. While plants are not knowledge per se, the interaction of humans and agricultural plants functions somewhat like a knowledge-based system. Plant breeders continue to select for improved traits and to build on the work of farmers and plant breeders that has occurred since the dawn of agriculture some 10,000 years ago. In this way, restricting access to plant germplasm prevents others from continuing to use that accumulated “knowledge” to select improved genotypes. For example, many new lettuce cultivars are being patented. This includes patents on cultivars, such as “Multigreen 57” lettuce [5], that prevent others from using that cultivar for any purpose without first obtaining a license from the patent holder (Fig 1). This is in contrast to a cultivar or line, such as “SM13-R2,” released into the public domain by the US Department of Agriculture lettuce breeding program in California [6]. While USDA lines are freely available to use, the resulting hybrid from a cross with this line could be proprietary. In contrast, cultivars released under the Open Source Seed Initiative (OSSI) Pledge, such as the open-pollinated “Chartreuse Butter Tongue” cultivar developed by Frank Morton, can be used freely for any purpose on the condition that all derivatives and seed retain the same freedoms (Fig 1). While many broad-based utility patents are still pending, plant breeders who have developed cultivars with similar traits may be unable to use them for 20 years if the utility patents are ultimately granted. Additionally, “bag tag” licenses attached to seed packets and certain restrictive contracts required for seed purchases contain provisions that prevent the saving and reuse of seed, essentially creating a “one-time rental” agreement for use of seed [7]. Contracts may also inhibit use of seed for research purposes in the public sector if, for example, the details of the contract restrict publication of data.

### The Open Source Seed Initiative

Concerns over the difficulties of accessing genetic resources in the face of increasing use of restrictive IPRs led a group of plant breeders, farmers, nonprofit agencies, seed advocates, and policymakers to create the nonprofit organization: the Open Source Seed Initiative (OSSI). Following open source principles, OSSI seeks to maintain fair and open access to plant genetic resources worldwide to foster innovative plant breeding, develop productive and resilient cultivars, and provide an alternative to other forms of IPR.

Critical to supporting these goals is the use of an open source mechanism for creation of a protected commons [9] for crop plant germplasm that fosters exchange and innovation among farmers, plant breeders, seed companies, and consumers in a viral fashion without restrictions on further breeding. The idea of a protected commons was coined by Richard Jefferson of CAMBIA in an effort to integrate biotechnology and biological research with “copyleft” licensing arrangements that “support both freedom to operate, and freedom to cooperate” in a “protected commons” [10]. A protected commons allows for public access and improvement of a resource without imposing restrictions on downstream use. OSSI created a pledge that embraces this approach. Users that accept the OSSI Pledge agree that no further restrictions can be made. OSSI promotes farmers’ and gardeners’ rights to save and replant seed of crops grown on their land and open access to plant germplasm for breeding. While both of these values are also shared by the Union for the Protection of New Varieties of Plants (UPOV) and the US Plant Variety Protection Act (PVPA) (Table 1), what distinguishes OSSI from either UPOV or PVPA is that no restrictions can be placed on derivative use of seeds distributed under the OSSI Pledge. Thus, OSSI-Pledged seeds can be bred, sold, shared, and reproduced as long as any subsequent derivative or reproduction of that seed also carries the same freedoms.

OSSI initially developed a legal license similar to the “copyleft” licenses of the free and open-source software movements [7] to accomplish the task of developing a protected commons for plant germplasm. However, after consultation with many plant breeders and seed companies, the eight-page license proved too cumbersome for practical use. Consequently, the OSSI Pledge was developed as a social mechanism rather than a legal license to create a protected germplasm commons and encourage unfettered exchange of seed now and into the future [11]. The OSSI Pledge reads:

“You have the freedom to use these OSSI-Pledged seeds in any way you choose. In return, you pledge not to restrict others’ use of these seeds or their derivatives by patents or other means, and to include this pledge with any transfer of these seeds or their derivatives.”

OSSI is primarily an education and outreach organization, serving as a collection point for open-source plant cultivars and developing a community of users and contributors. Plant breeders who have bred novel cultivars and who would like to release them as OSSI-Pledged are able to submit that information to the OSSI database, where they are reviewed by a subcommittee of OSSI board members. After they are accepted, OSSI then lists OSSI-Pledged cultivars and the seed companies that sell them on its website. Plant breeders situated in a wide variety of locations—from universities to freelancers to farmer-breeders to seed companies—have found that the OSSI Pledge and social support of the OSSI community provides the type of release mechanism that ensures sharing of cultivars as well as recognition of their plant breeding efforts. OSSI is not a seed company, but partners with seed companies who are selling OSSI-Pledged cultivars. Seed companies see an advantage to participating in the community because OSSI creates business for their companies by directing those interested in purchasing open-source seed to their websites. OSSI now has over 250 cultivars, contributed by over 28 plant breeders and sold by over 33 different companies.

Open systems are characterized by peer-based production that uses social mechanisms to achieve cooperation. Inherently, there is participation from a diverse set of actors with wide-ranging motivations and from various institutional contexts [12]. Additionally, instead of controlling users entirely through direct payment incentives, open systems rely on non-market-based incentives, both extrinsic (such as enhancing reputation and developing social networks) and intrinsic (such as creating a sense of social belonging) [13]. OSSI is creating a system for open exchange of plant germplasm by (1) encouraging dissemination of OSSI-Pledged cultivars and all derivatives of OSSI-Pledged cultivars, thus creating a protected commons of plant germplasm; (2) maintaining access to plant germplasm and the ability to share that germplasm freely with others; (3) fostering a decentralized and innovative plant breeding system; and (4) crediting the source and providing equitable benefit sharing so that plant breeders and stewards of plant germplasm receive recognition and potentially monetary rewards for their efforts.

The OSSI database and website provide recognition to plant breeders and credits them for the effort that they have put into creating improved cultivars. As funding for public plant breeding has declined, new sources of revenue to support plant breeding programs are needed. OSSI is interested in ensuring equitable benefit sharing and creating novel mechanisms for funding plant breeding activities. For example, a plant breeder who releases a cultivar under the OSSI Pledge can make royalty-like arrangements with a seed company interested in selling the seed of that cultivar commercially, as long as the arrangement is binding only to the two contracting parties and does not limit further use of that seed. OSSI does not seek to control those arrangements between a breeder and a seed seller as long as they do not restrict the ultimate recipient of the seed in any way. In addition, OSSI is working on several innovative partnerships between food stores, seed companies, farmers, and plant breeders to develop mechanisms to support plant breeding by generating awareness of open-source genetics at the consumer level.

Creating an open-source system for plant germplasm has the potential for many positive effects. The genetic diversity contained within seeds is essential for healthy farming systems and food security. Humans have been selecting for desirable traits and adapting plants to different environments using plant breeding techniques since the beginning of agriculture. Plant breeding, whether by farmers or breeders, can select genetics that most complement sustainable systems. Farmers and breeders must have access to diverse plant genetic resources in order to ensure that plants adapt to new pests and changing environmental conditions. Through utilization of an open-source pledge, OSSI is creating an ethical sharing and distribution mechanism for plant breeding. The enthusiasm by plant breeders, seed companies, farmers, and gardeners demonstrates support for the type of ethical value chain and sharing economy that OSSI embodies. The creation of a community that includes all players in the seed system has been critical to the success of this initiative.

## Abbreviations

IPR: intellectual property rights
MTA: material transfer agreement
OSSI: Open Source Seed Initiative
PVPA: Plant Variety Protection Act
UPOV: Union for the Protection of New Varieties of Plants
